# Optimizing 3D Convolution Kernels on Stereo Matching for Resource Efficient Computations

**DOI:** 10.3390/s21206808

**Published:** 2021-10-13

**Authors:** Jianqiang Xiao, Dianbo Ma, Satoshi Yamane

**Affiliations:** Division of Electrical Engineering and Computer Science, Kanazawa University, Kanazawa 920-1192, Japan; kxiao@csl.ec.t.kanazawa-u.ac.jp (J.X.); dma@csl.ec.t.kanazawa-u.ac.jp (D.M.)

**Keywords:** stereo matching, lightweight 3D kernels, 3D channel-wise attention, network design, 3D vision

## Abstract

Despite recent stereo matching algorithms achieving significant results on public benchmarks, the problem of requiring heavy computation remains unsolved. Most works focus on designing an architecture to reduce the computational complexity, while we take aim at optimizing 3D convolution kernels on the Pyramid Stereo Matching Network (PSMNet) for solving the problem. In this paper, we design a series of comparative experiments exploring the performance of well-known convolution kernels on PSMNet. Our model saves the computational complexity from 256.66 G MAdd (Multiply-Add operations) to 69.03 G MAdd (198.47 G MAdd to 10.84 G MAdd for only considering 3D convolutional neural networks) without losing accuracy. On Scene Flow and KITTI 2015 datasets, our model achieves results comparable to the state-of-the-art with a low computational cost.

## 1. Introduction

Stereo matching plays an important role in 3D computer vision applications, such as augmented reality (AR) [1], mixed reality (MR) [2], autonomous vehicle [3] and robot navigation [4,5]. It provides accurate disparity by a pair of stereo images. We can calculate the depth value by D=fB/d, where *d* denotes the disparity of the pixel, *f* is the focal length of the camera and *B* is the distance between the camera centers [6]. To get a precise disparity map is one of the most important tasks in stereo vision.

Classic stereo matching algorithms contain four parts: matching cost computation, cost support aggregation, disparity computation and disparity optimization [7]. Early studies perform machine learning methods to optimize disparity by Markov random field [8], conditional random field [9] or random forest [10]. With the rise of convolutional neural networks (CNNs), CNN-based approaches have been developed progressively. MC-CNN [6] first investigates CNNs on matching corresponding points for disparity estimation. Geometry and Context Network (GC-Net) [11] makes the training process end-to-end with a differentiable ArgMin operation on disparity estimations. The Pyramid Stereo Matching Network (PSMNet) [12] introduces spatial pyramid pooling and a stacked hourglass module for an accurate disparity map. These famous studies form a CNN-based approach framework: 2D Siamese feature extraction, cost volume aggregation, cost volume regularization and disparity regression.

One major problem with current CNN-based stereo matching algorithms is the enormous computation for cost volume regularization. The cost volume aggregation stage builds correspondence between left and right feature maps, aggregating disparity as an additional dimension on left feature maps to form 4D cost volumes [11]. For the cost volume regularization stage, most CNN-based methods build 3D convolution layers and 3D deconvolution layers, composing a 3D encoder–decoder architecture for regularizing 4D cost volumes. Compared to 2D convolution kernels, the additional dimension forming 3D convolution kernels raises computation complexity exponentially, leading the cost volume regularization to contain most of the computation among the entire architecture. Therefore, we build the 3D version of resource efficient kernel-based methods which can match up with the logic of a 3D encoder–decoder. By classifying all 3D layers in PSMNet according to their functions, we replace the original layers with our lightweight 3D convolution layers and implement a series of comparative experiments. Eventually, compared to the original PSMNet, we save 34.3% parameters and 73.1% multiply-add operations (MAdd) (95.50% paramters and 94.53% MAdd for 3D CNNs) without losing performance. We evaluate our model on Scene Flow and KITTI 2015 public datasets and obtain competitive results with other state-of-the-art stereo matching algorithms.

Our main contributions are listed below:

- A throughout discussion about optimizing already established 3D convolution kernels on stereo matching algorithms;
- A network design guideline when optimizing 3D convolution kernels on stereo matching algorithms for accurate disparity estimation and less computational complexity;
- By following the guideline above and without changing the architecture, our model performs comparable results to modern stereo matching algorithms with significantly less computational complexity.

## 2. Related Works

### 2.1. Kernel-Based Methods

Plenty of recent studies focus on building lightweight convolution kernels, which comprise the CNN-based application suitable for low resource devices, such as mobile phone and navigation robot. The origin SqueezeNet [13] achieves AlexNet [14]-level accuracy with 50 times fewer parameters. Xception [15] and MobileNetV1 [16] implement depthwise separable convolutions for reducing the model parameters. MobileNetV2 [17] proposes an inverted residual block with channel expansion for boosting the performance. ShuffleNetV1 [18] presents grouped pointwise convolution and a channel shuffle operation to save computation. ShuffleNetV2 [19] further considers the relationship between hardware and network design, improving their performance in terms of speed and accuracy.

The channel-wise attention mechanism has proven the potential for enhancing the performance with a small implementation. Squeeze and excitation networks (SE-Net) [20] firstly present an effective attention mechanism by aggregating a feature map with global average pooling along the channel and weights it on the respective channel. Selective kernel networks (SK-Net) [21] improve the performance with optimizing the channel-wise information in two different sizes of receptive fields. Efficient channel attention networks (ECA-Net) [22] propose an effective channel attention module for saving computational burden.

### 2.2. Stereo Matching

GC-Net [11] settles the basic framework of end-to-end deep learning-based stereo matching algorithms: 2D Siamese CNNs, cost volume aggregation, cost volume regularization and disparity regression. Zhou et al. [23] draw a review of deep learning-based methods on stereo matching algorithms, which shows that recent stereo matching algorithms follow this framework and improve network performance by modifying partially with other mechanisms. We explain the main recent works according to where they are modified in Table 1.

According to Table 1, most of the works focus on optimizing the network architecture, especially the cost volume aggregation part, which costs the greatest computational resources. Yet, 3D convolution layers and 3D transposed convolution layers, the basic elements of cost volume aggregation, have been rarely studied. For exploring the limitation of the 3D kernel-based method on stereo matching methods, inspired by [39], we conduct a complete investigation optimizing network architecture with 3D kernel-based methods. We perform PSMNet as the basic architecture, attempting an elaborate study on all stages of the cost volume normalization with 3D kernel-based methods. By analyzing the model’s complexity and accuracy on Scene Flow and the KITTI 2015 data set, we receive a model with comparable results and less computational complexity.

## 3. Network Architecture

We first introduce the details about our network structure, including the architecture of the prototype PSMNet [12], and a series of 3D convolution kernels as the basic components of the network design pipeline.

### 3.1. PSMNet

**Figure 1:**
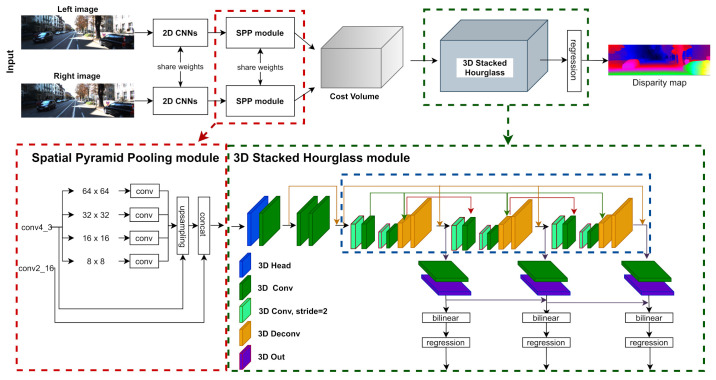
Architecture of PSMNet: We specified all 3D CNN kernels with different colors representing different interactions on the size and channel of cost volumes.

In our paper, we take the PSMNet as the baseline and explore a series of 3D kernel-based methods to find a lightweight and accuracy model. In Figure 1, we separate all 3D convolution kernels into five categories: 3D head, 3D convolution, 3D convolution with stride=2, 3D deconvolution and 3D output. Table 2 shows the network settings of PSMNet, we optimize the network design by experimenting with the 3D kernel-based method on different stages of the architecture.

The 4D cost volumes (disparity×height×width×channel) are formed by concatenating the left and the right feature maps $f_{l},f_{r}$ (height×width×channel) in Equation (1):

(1) $$C\left(d,x,y,channel\right)=\left(Concatf_{l}\left(x,y\right),f_{r}\left(x-d,y\right),channel\right).$$

The 3D stacked hourglass module performs cost volume regularization. The continuous disparity map is obtained by the disparity regression process in [11]. The output disparity $\hat{d}$ is calculated as the summation of each disparity *d* weighted by corresponding probability $\sigma (-c_{d})$,

(2) $$\hat{d}=\sum_{d=0}^{D_{max}}d\times \sigma \left(-c_{d}\right).$$

The σ(.) denotes softmax operation, and the maximum disparity $D_{max}$ is set to 192.

For generating a smooth disparity map, the PSMNet uses a smooth L1 loss function to train the whole architecture. The loss function of PSMNet is defined as:

(3) $$L\left(d,\hat{d}\right)=\frac{1}{N}\sum_{i=1}^{N}smooth_{L1}\left(d_{i}-{\hat{d}}_{i}\right),$$

where

(4) $$smooth_{L1}\left(x\right)=\left\{\begin{array}{cc} 0.5x^{2}, & if\;|x|<1\\ |x|-0.5, & otherwise \end{array}\right..$$

*N* is the amount of label pixels. *d* and $\hat{d}$ are the ground-truth disparity and predicted disparity, respectively.

### 3.2. Architecture of 3D Convolution Kernels

In this section, we introduce the architecture of our 3D kernel-based methods. We build all 3D kernels based on their 2D version and fit them to the 3D part of PSMNet according to categories of layers in Figure 1.

#### 3.2.1. 3D MobileNetV1

**Figure 2:**
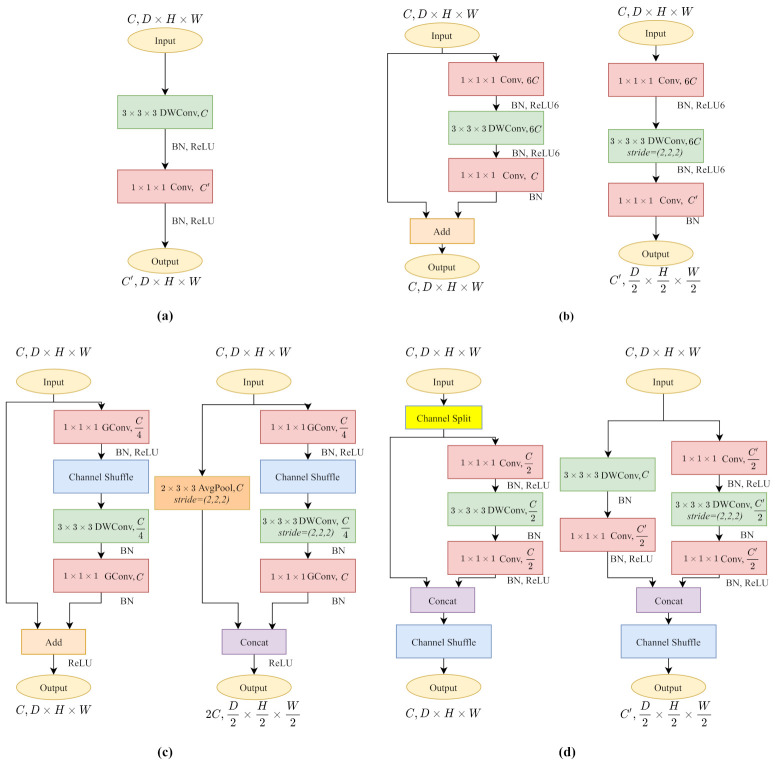
The architecture of 3D lightweight CNN kernels. (**a**) 3D MobileNetV1. (**b**) 3D MobileNetV2. (**c**) 3D ShuffleNetV1. (**d**) 3D ShuffleNetV2. Each module has the same effect as a 3×3×3 convolution kernel.

As shown in Figure 2a, 3D MobileNetV1 [16] decomposes a standard 3×3×3 convolution kernel into a 3×3×3 depthwise separable convolution and a 1×1×1 pointwise convolution. The 3D depthwise separable convolution exploits a series of convolutional filters according to the channel number of the input cost volume and extracts local context in a channel-wise manner. Pointwise convolution walks through the cost volumes, restoring spatial information across different channels.

By isolating local context extraction and channel interaction, MobileNetV1 decreases computational complexity and model size significantly. Unlike most recent CNN architecture, MobileNetV1 excludes ResNet-like residual connections [40] or multi-branch operations, which makes it accessible for channel-wise operations (3D Head in Table 2).

#### 3.2.2. 3D MobileNetV2

Figure 2b shows the 3D MobileNetV2 [17] block. It follows the main idea of MobileNetV1 by building depthwise convolution layers and pointwise convolution layers for reducing computational complexity. It also proposes inverted residual blocks with linear bottlenecks and residual connections. The linear bottlenecks increase the cost volume channels with an expansion factor for solving the problem that high dimensional targeting feature expression often collapses when operating the rectified linear unit (ReLU) activation. The 3D MobileNetV2 block with stride=1 (Figure 2b-left) comprising the inverted residual structure helps to construct a deeper model as ResNet [40], while the block with stride=2 (Figure 2b-right) keeps excluding the residual connections for a smooth channel-wise operation.

#### 3.2.3. 3D ShuffleNetV1

Compared to other lightweight CNNs, ShuffleNetV1 [18] uses 1×1×1 pointwise group convolutions (GConv) for computational efficiency. As shown in Figure 2c, the symbolic channel shuffle operation helps to break through the barriers of different groups to build a more robust model. Unlike MobileNetV2, ShuffleNetV1 follows the residual structure to decrease the feature map channels to make it lightweight.

The 3D ShuffleNetV1 block with stride=1 (Figure 2c-left) builds the standard ResNet-like residual connections, while the stride=2 version constructs the residual connections in another way. As shown in Figure 2c-right, the main branch keeps the structure unchanged, downsamples the feature maps by half with a strided depthwise convolution (DWConv). On the other hand, the shortcut branch utilizes average pooling to halve the feature maps. As the output feature channels of two branches are *C*, the concatenation results raise feature channels to 2C.

#### 3.2.4. 3D ShuffleNetV2

Compared to ShuffleNetV1, ShuffleNetV2 [19] changes the 1×1×1 pointwise group convolution into standard pointwise convolution. In Figure 2d, since the pointwise convolution is not grouped, the channel shuffle operation is placed after the two-branches concatenation to enable information communication between two branches.

The 3D ShuffleNetV2 block with stride=1 (Figure 2d-left) shuffles the feature channels and splits all feature maps by two with a channel split operation. Half of them remain untouched with the residual connection. Another half follows a three convolutions scheme without changing the channels. The stride=2 version (Figure 2d-right) makes use of all feature maps on each branch. Commonly, the down sampling layers contain channel increases. The main branch (right) accomplishes the channel variation on the first pointwise convolution. However, the identity branch compiles the channel change after the 3×3×3 depthwise convolution to keep the channel-wise dependency until the pointwise layer.

#### 3.2.5. 3D ECA Blocks

**Figure 3:**
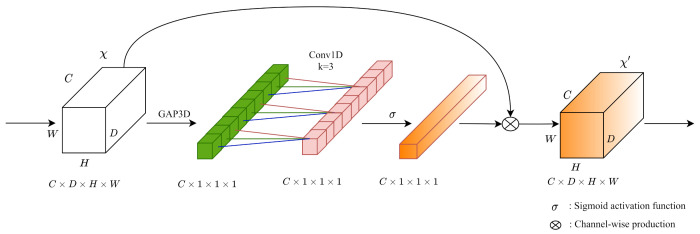
The architecture of 3D ECA blocks. We build the 3D ECA blocks for exploring channel-wise attention during cost volume regularization. In the original paper, the kernel size *k* of Conv1D is adaptively determined according to channel dimension *C*. In the 3D part of PSMNet, we set all kernel sizes *k* to 3 in terms of the relatively small feature channels.

For the outputting cost volume followed by 3D CNNs, we build the 3D ECA [22] blocks for optimizing channel-wise attention. Figure 3 shows that our 3D ECA blocks aggregate cost volumes (D×H×W) along the channel with a 3D global average pooling operation. Different from other channel-wise attention modules, ECA blocks do not perform dimensionality reductions when extracting channel-wise information with 3D global average pooling. Then 1D convolution achieves information aggregation on the nearby extracted channel information. After passing a Sigmoid activation function (σ), we implement the channel-wise product on the input cost volume to form a 3D channel-wise attention mechanism.

## 4. Computational Complexity Matrics

Before we dive into the network design pipeline, we introduce the metrics for evaluating the computational complexity as follows:

- Parameters are the number of trainable neurons in the designed convolutional neural network;
- Multiply-Add operations(MAdd) describe the accumulated operations when training neural networks. [41] explain the calculation of floating point operations (FLOPs). MAdd is approximately half of FLOPs;
- Memory Access Cost (MAC) is the amount of allocating computational resource during the training process;
- Model Size shows the storage size of all trained parameters.

## 5. Network Design Pipline

In this chapter, we use the 3D kernel-based methods introduced above to replace the standard 3D CNNs in PSMNet, and design a series of comparative experiments to illustrate the impact of different 3D convolution kernels on the performance. Since we focus on discussing the role of 3D convolution kernels in the stereo matching algorithm, we completely follow the original PSMNet on the network layer setting in Table 2.

### 5.1. Implementation

For all models, we use the same implementation for a fair comparison.

#### 5.1.1. Dataset and Evaluation Metrics

We evaluate our models on Scene Flow [42] and KITTI 2015 [43] datasets:

- Scene Flow is a large scale dataset with synthetic stereo images. It contains 35,454 training and 4370 testing image pairs with 940×540 resolutions. We report the end-point-error (EPE) for evaluations, where EPE shows the average disparity error in pixels;
- KITTI 2015 contains real street scenes taken by driving a car. It includes 200 training image pairs with ground truth disparity maps collected by LiDAR and 200 other test image pairs without ground truth disparity. The size of the training and test images is 1240×376. We repot D1-all metrics as the official leaderboard.

#### 5.1.2. Implementation Details

We train all models with an Adam optimizer on one NVIDIA RTX 3090 GPU. During the training process, all input images are randomly cropped to 512×256. We first train our models from scratch on the Scene Flow dataset for 20 epochs with a batch size of four. The learning rate is 0.0005 constantly. Then we train the models on KITTI 2015 with Scene Flow pre-trained weights for 2000 epochs. The initial learning rate is 0.0005 and is decreased by half at 400th, 600th and 800th epochs. Since the training dataset of KITTI 2015 only contains 200 input pairs, we perform the training process with 10-fold cross validation [44] for preventing overfitting. In Appendix A, we discuss the difference between normal cross validation and 10-fold cross validation during the re-implementation of the original PSMNet on KITTI 2015 dataset.

### 5.2. Optimize 3D Convolution Kernels in PSMNet

In Figure 1 and Table 2, we specify all 3D convolution kernels in PSMNet to five categories: 3D Head, 3D Conv, 3D Conv stride=2, 3D Deconv and 3D Out. We replace the traditional CNNs in PSMNet with the 3D convolution kernels in Section 3.2 according to different functions of 3D convolution kernels.

#### 5.2.1. 3D Head

The first 3D convolution layer (3D Head) reduces the number of channels of the cost volume from 64 to 32. Among all 3D convolution kernels, MobileNetV1 builds without residual connection, which is beneficial when operating feature channels. We build 3D MobileNetV1 on the first layer.

#### 5.2.2. 3D Convoltion Layers

For all 3D convolution layers (3D Conv and 3D Conv stride=2), we used MobileNetV1, MobileNetV2, ShuffleNetV1 and ShuffleNetV2 to replace the original kernels.

**Figure 4:**
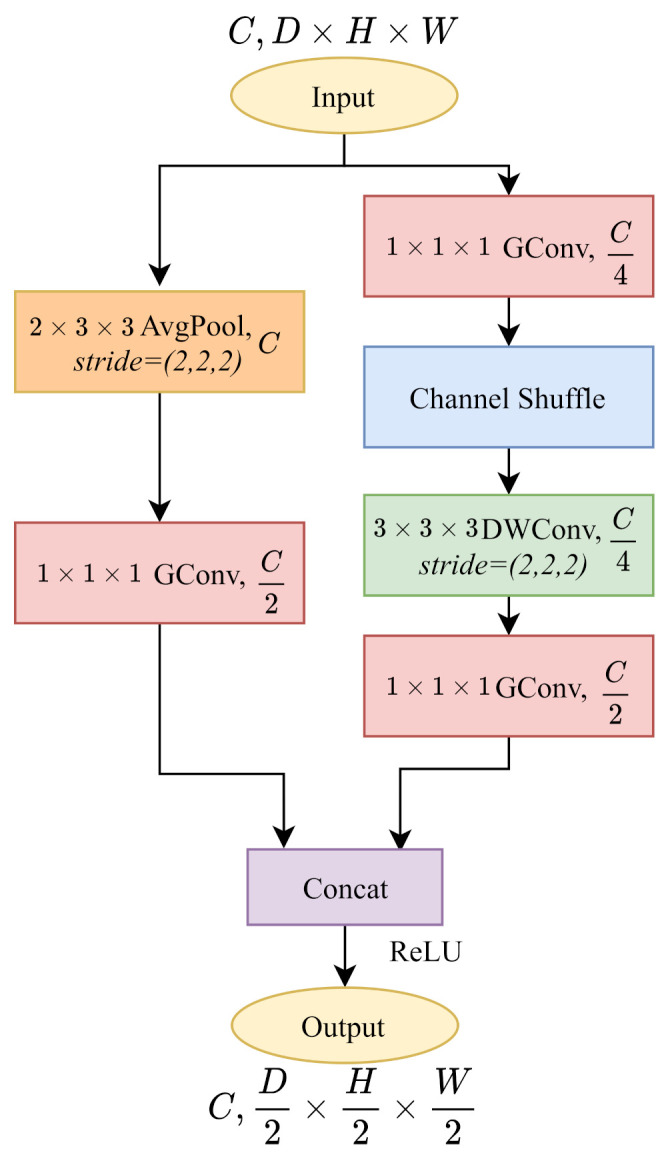
The implementation of 3D ShuffleNetV1 with stride=2 at 3DStack3_x layer. Following the PSMNet, we downsample the cost volume by half without changing the channels.

In Figure 2c-right, ShuffleNetV1 with stride=2 always blocks double the channels to 2C. However, as shown in Table 2, 3DStack3_x layer downsamples the cost volume without changing the channels. To make an impartial comparison with PSMNet, as shown in Figure 4, we build a ShuffleNetV1 block with downsampling without changing the number of output channels. In the main branch, we modify the channel of the last pointwise group convolution into $\frac{C}{2}$. As for the identity branch, we add a pointwise group convolution followed by the 3D average pooling layer and decrease the channels to $\frac{C}{2}$. Therefore, the output cost volumes remain with the same channel number as the PSMNet architecture.

**Figure 5:**
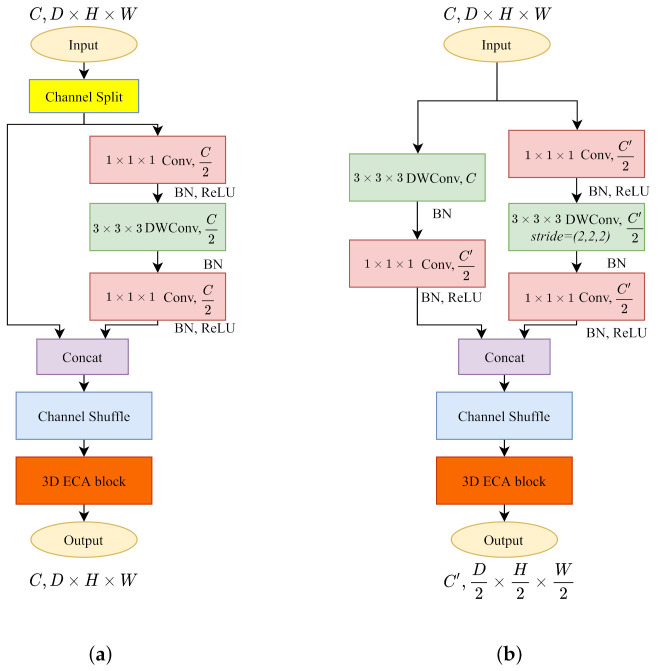
We build the 3D ECA blocks after the channel shuffle operations on 3D ShuffleNet V2. (**a**) 3D ECA-ShuffleNetV2 with stride=1. (**b**) 3D ECA-ShuffleNetV2 with stride=2.

After obtaining the results in Table 3 through comparative experiments, we found that 3D ShuffleNetV2 performs the best among all 3D convolution kernels. Then, we added the 3D ECA block on 3D ShuffleNetV2. The original paper only implements the ECA block on residual connection modules. However, the concatenation in ShuffleNetV2 and the addition operation in the residual connection reflect different logic when operating the channels of cost volumes. We discussed the most suitable position for inserting a 3D ECA block in ShuffleNetV2 in Appendix B. The 3D ECA-ShuffleNetV2 is shown in Figure 5.

#### 5.2.3. 3D Deconvoltion Layers

Three dimensional (3D) transposed convolutions upsample the input cost volumes to twice the size. It first expands the input size by zero padding the cost volume of each channel, and then compiles the standard 3D convolution to introduce learning parameters for more refined textural information. In our implementation, we follow the ShuffleNetV2 as the 3D convolution. However, when inserting 0 values to restore the size, the pointwise convolution will destroy the learned semantic information and textural information. We add upsamping with trilinear interpolation before ShuffleNetV2 for a continuous and rough cost volume, then the ShuffleNetV2 block restores the cost volume with a series of parameterized convolution layers.

**Figure 6:**
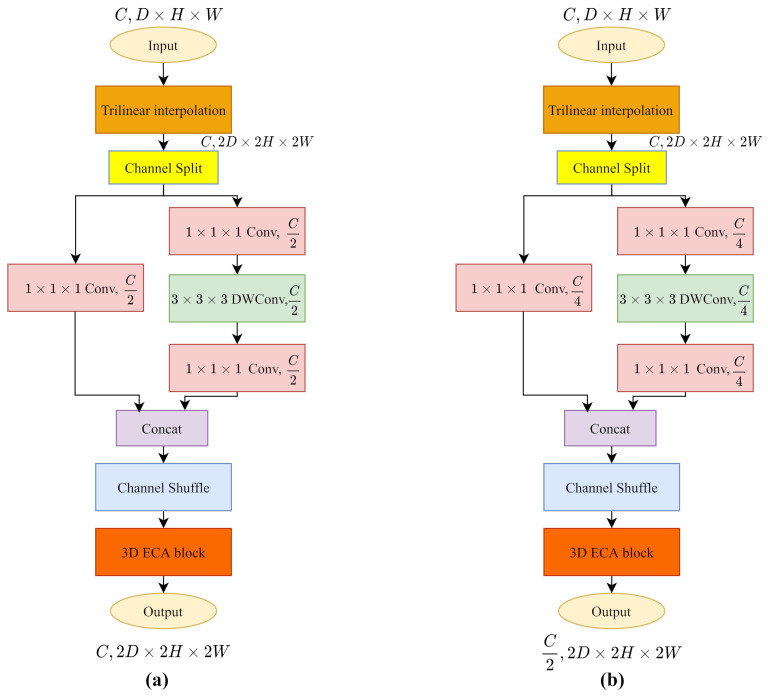
The trilinear interpolation upsamples the input to roughly twice the size of cost volume, then the 3D ECA-ShuffleNetV2 refines the resolution of cost volume with parameterized layers. (**a**) 3D Transposed ECA-ShuffleNetV2 at 3DStack5_x layer. (**b**) 3D Transposed ECA-ShuffleNetV2 at 3DStack6_x layer.

Following the architecture of PSMNet in Table 2, we build the 3DStack5_x layer by simply adding an upsampling layer as we mentioned. Since the channel needs to be reduced to half, we construct the 3DStack6_x layer as illustrated in Figure 6. The channel split operation divides the input channels into two branches. In the main branch (right), the first pointwise convolution decreases the channels to $\frac{C}{4}$ instead $\frac{C}{2}$. In the identity branch (left), we include a pointwise convolution to reduce the channels to $\frac{C}{4}$. Eventually, we get the output cost volume with twice the size and half the channels.

#### 5.2.4. 3D Out

For the last 3D CNN layer (Out2_x), we keep the 3D convolution kernel unchanged for establishing the disparity and textural information.

#### 5.2.5. Network Design Overview

As shown in Table 3, in the first stage, we discuss the influence of MobileNetV1, MobileNetV2, ShuffleNetV1 and ShuffleNetV2 as the basic 3D convolution kernels of the PSMNet on the accuracy and computational complexity. Then we follow the same strategy for designing comparative experiments. We put the MobileNetV1 on the first layer for saving computation, ECA blocks on every 3D convolution kernel for boosting the model accuracy and transposed ShuffleNetV2 for switching all 3D parts of PSMNet to a lightweight method. Eventually, the computational complexity (MAdd) of PSMNet reduced from 256.66 G to 69.03 G 3D convolution kernels implementation. Parameters and model size also decrease from 5.23 M to 3.43 M, and from 21.1 M to 14.1 M, respectively. MAC increases to build more small layers during training. We reduced the computational complexity of the model while keeping the performance almost unchanged. In Appendix C, we parameterize of all models in a layer-manner to emphasize how different 3D convolution kernels change the model computational complexity.

## 6. Benchmark Results

In Section 5, we built 3D convolution kernels and explored the best combination of 3D kernels with comparative experiments. Due to the number of comparative experiments being relatively large, for saving time, we only train all models to close results without convergence. For benchmarking, we train our model on two NVIDIA V100 for setting the batch size to eight. Since now we have a larger batch size, we double the learning rate for two stages of training. For Scene Flow, the learning is 0.001 constantly. For KITTI 2015, the initial learning rate is 0.001 and is decreased by half at the 400th, 600th and 800th epochs. We perform 10-fold cross validation on the first 1000 epochs. Then we train another 1000 epochs without 10-fold cross validation with a 0.000125 learning rate.

**Figure 7:**
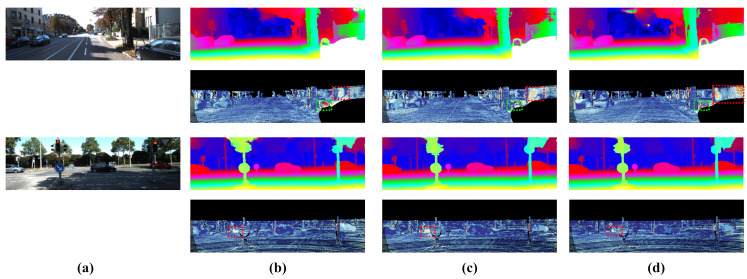
Visualization of prediction error on KITTI test set (red and yellow pixels denote error disparities). The red boxes denote foreground regions and green boxes denote background regions. (**a**) Left image. (**b**) Our model. (**c**) PSMNet. (**d**) AANet.

We evaluate our model on Scene Flow and KITTI 2015. In Table 4, our model achieves accurate results on these datasets with a low-cost MAdd in terms of computation. For Scene Flow, our model outperforms the original PSMNet 0.18 on end-point-errors. As for KITTI 2015, Table 5 demonstrates that our model achieves similar results to PSMNet, only taking 26.9% of the MAdd. Meanwhile, our model surpasses the PSMNet significantly in foreground pixels (D1-fg in the table). Figure 7 further visualizes the disparity estimation result on the KITTI 2015 test set.

## 7. Discussion

In Table 5, compared to other studies, our model contains very little computational complexity. However, the runtime is longer than that of the original PSMNet. As mentioned in [47,48], the reason may be that the cuDNN library does not fully support depthwise convolutions and pointwise convolutions. For the GPU platform of the cuDNN library, the optimization of classic convolutions on end-to-end training is better. So, it will be faster than some lightweight convolutions, although it produces more computation theoretically. We present the runtime of all models in Table 6.

## 8. Conclusions

In this paper, based on PSMNet as a prototype, we design a series of kernel-based methods aiming for a lightweight and accurate model without modifying the original architecture. By optimizing 3D convolution kernels with corresponding kernel-based methods, our model greatly reduces computational complexity and achieves comparable results to the modern stereo matching algorithms. In future work, as we mentioned in Section 7, we will investigate the implementation of 3D depthwise convolutions and 3D pointwise convolutions on the cuDNN library and improve our model to become faster in training and inference.

## Figures and Tables

**Table 1:** Recent studies on stereo matching. We clarify where their modifications target stereo matching architecture.

Name	Main Feature	Targeting Part
GwcNet [24]	Performs a group-wise correlation to generate	Cost volume aggregation &
	a multi-feature cost volume.	Cost volume regularization
StereoNet [25]	Utilizes more downsampling to form a low-resolution cost volume,	2D Siamese CNNs &
	which lead a great improvement in speed.	Cost volume regularization
GANet [26]	Introduces a semi-global guided aggregation layer and	Cost volume regularization
	a local guided aggregation layer	
	for replacing 3D convolution layer in 3D encoder-decoder.	
Zhu et al. [27]	Apply edge-preserving guided-Image-filtering (GIF) at	Cost volume regularization
	different resolutions on multi-scale stereo matching.	
AcfNet [28]	Includes the ground truth cost volume and	Cost volume regularization
	confidence map for intermediate supervision.	
DeepPruner [29]	Aggregates a sparse cost volume	Cost volume aggregation
	with a differentiable PatchMatch [30] module.	
AANet [31]	Presents a intra-scale aggregation module	Cost volume regularization
	for replacing 3D convolution layer & cross-scale aggregation for	
	integrating multi-scale cost volume.	
CSN [32] & CFNet [33]	Implements the architecture in a coarse-to-fine manner.	Cost volume aggregation &
		Cost volume regularization
Wei et al. [34]	Improve StereoNet[25] with edge-guided refinement &	2D Siamese CNNs &
	multi-cross attention module on multi-level cost volumes.	Cost volume regularization
Huang et al. [35]	Implement ResNetXt [36] and Atrous Spatial Pyramid Pooling	2D Siamese CNNs
	(ASSP) [37] on 2D CNNs.	
JDCNet [38]	Use the 2D stereo encoder-decoder to generate a disparity range	2D Siamese CNNs &
	for guiding 3D aggregation network.	Cost volume aggregation

**Table 2:** Parameters of PSMNet architecture. Batch normalization and ReLU layers are used except the summation operations. *H* and *W* denote the height and width of an input pair. *D* means the disparities of the stereo input images.

	2D Part: Feature Extraction			3D Part: Cost Volume Optimization	
**Layer Name**	**Setting**	**Output Dimension**	**Layer Name**	**Setting**	**Output Dimension**
Stereo input	-	$\left[\begin{array}{c} H\times W\times 3 \end{array}\right]\times 2$	Concat left and right feature maps	-	$\frac{1}{4}D\times \frac{1}{4}H\times \frac{1}{4}W\times 64$
	**2D CNNs**		**3D Stacked Hourglass module**		
Conv0_x	$\left[\begin{array}{c} 3\times 3,32 \end{array}\right]\times 3$	$\frac{1}{2}H\times \frac{1}{2}W\times 32$	3DConv0(3D Head)	3×3×3, 32	$\frac{1}{4}D\times \frac{1}{4}H\times \frac{1}{4}W\times 32$
Conv1_x	$\left[\begin{array}{c} 3\times 3,32\\ 3\times 3,32 \end{array}\right]\times 3$	$\frac{1}{2}H\times \frac{1}{2}W\times 32$	3DConv1(3D Conv)	$\left[\begin{array}{c} 3\times 3\times 3,64 \end{array}\right]\times 3$	$\frac{1}{4}D\times \frac{1}{4}H\times \frac{1}{4}W\times 32$
Conv2_x	$\left[\begin{array}{c} 3\times 3,64\\ 3\times 3,64 \end{array}\right]\times 16$	$\frac{1}{4}H\times \frac{1}{4}W\times 64$	3DStack1_x(3D Conv, stride=2)	3×3×3,64,stride=2	$\frac{1}{8}D\times \frac{1}{8}H\times \frac{1}{8}W\times 64$
Conv3_x	$\left[\begin{array}{c} 3\times 3,128\\ 3\times 3,128 \end{array}\right]\times 3$	$\frac{1}{4}H\times \frac{1}{4}W\times 128$	3DStack2_x(3D Conv)	3×3×3,64	$\frac{1}{8}D\times \frac{1}{8}H\times \frac{1}{8}W\times 64$
Conv4_x	$\left[\begin{array}{c} 3\times 3,128\\ 3\times 3,128 \end{array}\right]\times 3,dila=2$	$\frac{1}{4}H\times \frac{1}{4}W\times 128$	3DStack3_x(3D Conv, stride=2)	3×3×3,64,stride=2	$\frac{1}{16}D\times \frac{1}{16}H\times \frac{1}{16}W\times 64$
	**Spatial Pyramid Pooling (SPP) module**		3DStack4_x(3D Conv)	3×3×3,64	$\frac{1}{16}D\times \frac{1}{16}H\times \frac{1}{16}W\times 64$
Branch_1	64×64, 128, Avg_pooling	$\frac{1}{4}H\times \frac{1}{4}W\times 32$	3DStack5_x(3D Deconv)	ConvTranspose3d 3×3×3,64	$\frac{1}{8}D\times \frac{1}{8}H\times \frac{1}{8}W\times 64$
	1×1, 32, Conv				
	Upsample, Biliner interpolation				
Branch_2	32×32, 128, Avg_pooling	$\frac{1}{4}H\times \frac{1}{4}W\times 32$	3DStack6_x(3D Deconv)	ConvTranspose3d 3×3×3,32	$\frac{1}{4}D\times \frac{1}{4}H\times \frac{1}{4}W\times 32$
	1×1, 32, Conv				
	Upsample, Biliner interpolation				
Branch_3	16×16, 128, Avg_pooling	$\frac{1}{4}H\times \frac{1}{4}W\times 32$	Out1_x(3D Conv)	3×3×3,32	$\frac{1}{4}D\times \frac{1}{4}H\times \frac{1}{4}W\times 32$
	1×1, 32, Conv				
	Upsample, Biliner interpolation				
Branch_4	8×8, 128, Avg_pooling	$\frac{1}{4}H\times \frac{1}{4}W\times 32$	Out2_x(3D Out)	3×3×3,1	$\frac{1}{4}D\times \frac{1}{4}H\times \frac{1}{4}W\times 1$
	1×1, 32, Conv				
	Upsample, Biliner interpolation				
Concat[Conv2_16,Conv4_3,Branch_1,Branch_2,Branch_3,Branch_4]		$\frac{1}{4}H\times \frac{1}{4}W\times 320$	Upsampling	Trilinear interpolation	D×H×W
Fusion	3×3, 128	$\frac{1}{4}H\times \frac{1}{4}W\times 32$	-	Disparity regression	H×W
	1×1, 32,				

**Table 3:** Experiments for 3D convolution kernels on PSMNet. We calculate the MAdd with 512×256 resolution as input. EPE is the end-point-error on the Scene Flow dataset. D1-all denotes the evaluation of the KITTI 2015 leaderboard. MobelNetV2* denotes implementation of the kernel with expansionratio=2 considering the MAC of the model. At each stage, we select the best 3D convolution kernel (grey line) and optimize it on the model.

3D Conv Kernel	EPE	D1-All	Parameters (Millon)	MAdd (Gb)	MAC (Mb)	Model Size (Mb)
**3D Convolution Layers**						
Baseline(re-implemented)	1.123	2.41	5.23	256.66	3895	21.1
MobileNetV1	1.252	2.68	3.97	168.96	5421	16.2
MobileNetV2*	1.215	2.60	4.11	175.93	6097	16.8
ShuffleNetV1	1.295	2.88	3.91	164.39	4651	16.0
ShuffleNetV2	1.241	2.62	3.96	168.03	5071	16.2
**3D Head**						
3D CNN	1.241	2.62	3.96	168.03	5071	16.2
MobileNetV1 (Head)	1.233	2.61	3.91	147.81	5411	16.0
**3D ECA Blocks**						
Without ECA Blocks	1.233	2.61	3.91	147.81	5411	16.0
ECA Blocks	1.160	2.50	3.91	147.92	5835	16.0
**3D Deconvoltion Layers**						
3D Transposed CNN	1.160	2.50	3.91	147.92	5835	16.0
Transposed ShuffleNetV2	1.124	2.43	3.43	69.03	6771	14.1

**Table 4:** Evaluation results on the Scene Flow dataset. Our model is competitive with other top-performing models.

Method	Ours	Baseline [16]	GC-Net [11]	GANet [26]	DeepPruner-Best [29]	DispNetC [42]	StereoNet [25]	JDCNet [38]
EPE	0.91	1.09	2.51	0.84	0.86	1.68	1.10	0.83

**Table 5:** Evaluation results on the KITTI 2015 dataset. The first seven methods are accurate methods (included the baseline). The other five are considered fast methods. Our model not only achieves results comparable to those of some accurate methods but also requires significantly less computational complexity. We only calculated the MAdd of some representative models.

Method	All (%)			Noc (%)			Runtime(s)	MAdd (G)
	D1-Bg	D1-Fg	D1-All	D1-Bg	D1-Fg	D1-All		
Baseline [12]	1.86	4.62	2.32	1.71	4.31	2.14	0.41	256.66
PSMNet-lite (Ours)	1.91	4.56	2.35	1.75	4.06	2.13	0.63	69.03
MC-CNN [6]	2.89	8.88	3.89	2.48	7.64	3.33	67	-
GC-Net [11]	2.21	6.16	2.87	2.02	5.58	2.61	0.9	733.36
GwcNet [24]	1.74	3.93	2.11	1.61	3.49	1.92	0.32	247.6
DeepPruner-Fast [29]	1.87	3.56	2.15	1.71	3.18	1.95	0.18	-
GANet-15 [26]	1.55	3.82	1.93	1.40	3.37	1.73	0.36	-
CSN [32]	1.59	4.03	2.00	1.43	3.55	1.78	0.6	-
SMD-Net [45]	1.69	4.01	2.08	1.54	3.70	1.89	0.41	-
StereoNet [25]	4.30	7.45	4.83	-	-	-	0.015	47.08
DispNetC [42]	4.32	4.41	4.34	4.11	3.72	4.05	0.03	-
DeepPruner-Best [29]	2.32	3.91	2.59	2.13	3.43	2.35	0.06	-
AANet [31]	1.99	5.39	2.55	1.80	4.93	2.32	0.062	-
Fast DS-CS [46]	2.83	4.31	3.08	2.53	3.74	2.73	0.02	-
JDCNet [38]	1.91	4.47	2.33	1.73	3.86	2.08	0.079	-

**Table 6:** Inference time of all models in the network design pipeline. We use one NVIDIA RTX 3090 for all implementations. “+ MobileNetV1” replaces the 3DConv0 with the 3D MobileNetV1 module. “+ ECA block” add 3D ECA block on all 3D kernels. “+ Transposed ShuffleNetV2” replaces the 3DStack5_x and 3DStack6_x deconvolution layers with 3D Transposed ECA-ShuffleNetV2.

Method	MobileNetV1	MobileNetV2	ShuffleNetV1	ShuffleNetV2	+ MobileNetV1	+ ECA Blocks	+ Transposed
					Head		ShuffleNetV2
Runtime (s)	0.48	0.53	0.35	0.40	0.41	0.60	0.63

**Figure A1:**
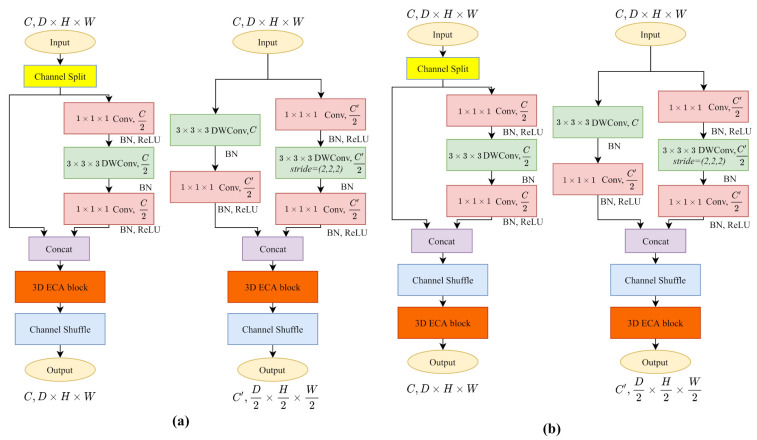
We built ECA block both before and after channel shuffle operation to find a more appropriate architecture of 3D ECA-ShuffleNetV2. (**a**) before channel shuffle operation. (**b**) after channel shuffle operation.

**Figure A2:**
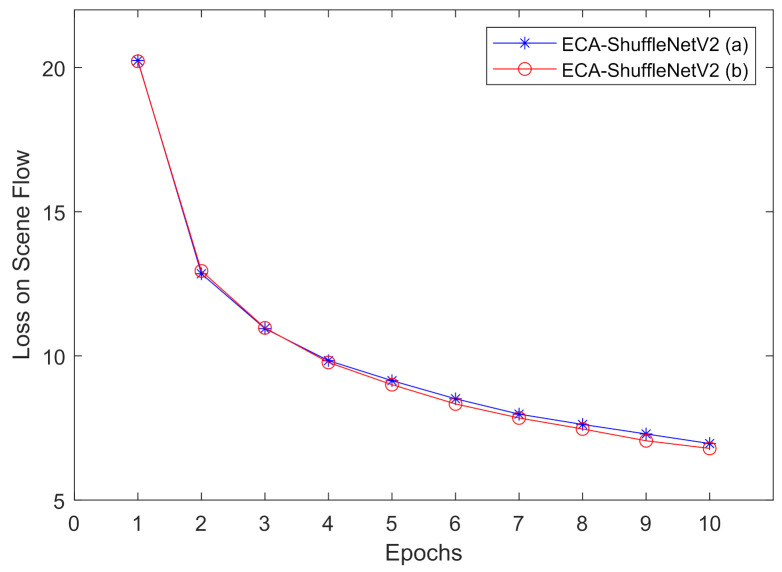
Training loss on Scene Flow data set with 3D ECA-ShuffeNetV2 (**a**) & (**b**).

## Data Availability

Scene Flow Datasets: FlyingThings3D, Driving, Monkaa in https://lmb.informatik.uni-freiburg.de/resources/datasets/SceneFlowDatasets.en.html (accessed on 16 September 2021); KITTI 2015 in http://www.cvlibs.net/datasets/kitti/eval_scene_flow.php?benchmark=stereo (accessed on 16 September 2021).

## Appendix A. 10-Fold Cross Validation

Since KITTI 2015 has a small amount of training set, during the re-implementation of PSMNet, we examine the normal training manner and 10-fold cross validation with the same pretrained model on Scene Flow data set.

**Table A1:** The re-implementation of PSMNet with cross validation and 10-fold cross validation. Both follow the same implementation details in Section 5.1.2. We calculate the average 3-pixel error of all image pairs in the whole training set with the model we submitted for evaluation.

Method	All (%)			Noc (%)			Loss	3-Pixel Error in
	D1-Bg	D1-Fg	D1-All	D1-Bg	D1-Fg	D1-All		Training Set
Cross Validation	2.03	4.89	2.51	1.86	4.53	2.30	0.291	0.707
10-fold Cross Validation	1.94	4.76	2.41	1.75	4.42	2.22	0.314	0.747

As illustrated in Table A1, the cross validation training manner leads to a lower loss and more accurate disparity map on the training set, while the evaluation result is worse. This situation reflects it has an explicit overfitting on the training set.

## Appendix B. 3D ECA-ShuffleNetV2 Blocks

In order to achieve the attention mechanism on each channel of the cost volumes, we build the 3D ECA block after concatenation of the main branch and the identity branch. Two potential 3D ECA-ShuffleNetV2 blocks are shown in Figure A1. For saving time, we only train both potential 3D ECA-ShuffleNetV2 blocks on Scene Flow data set for 10 epochs and evaluate the model by training loss.

We show the training performance in Figure A2, the (b) architecture slightly outperforms than (a) architecture. For model (a), after it learning the channel-wise attention information, the channel shuffle operation disrupts the channel order of cost volumes. Based on the results, we think that the order of channels contains part of the spatial representation ability of the model.

## Appendix C. Parameterize Model Details on Optimizing 3D Convolution Kernels

In order to reflect the influence of the reduction of computational complexity on building different 3D convolution kernels, we show the parameterize model details on Table A2. Since our work mainly focused on the regularization of the cost volume, we omit the model details of feature extraction to one output as 2DCNN. When only considering the 3D CNNs in the model, we reduce the parameters and MAdd from 1.887M to 0.085M (95.50%) and from 198.47G to 10.84G (94.53%).

**Table A2:** Parameterize model details on all 3D convolution kernels in our network design pipeline. The numbers in bold denote the modified layers compare to former stages.

Method	Basline		M_V1		M_V2		S_V1		S_V2		+M_V1_Head		+ECA		+Transposed S_V2	
Layer	Para(M)	MAdd(G)	Para(M)	MAdd(G)	Para(M)	MAdd(G)	Para(M)	MAdd(G)	Para(M)	MAdd(G)	Para(M)	MAdd(G)	Para(M)	MAdd(G)	Para(M)	MAdd(G)
2D CNNs	3.340	58.191	3.340	58.191	3.340	58.191	3.340	58.191	3.340	58.191	3.340	58.191	3.340	58.191	3.340	58.191
3DConv0	0.055	21.781	0.055	21.781	0.055	21.781	0.055	21.781	0.055	21.781	**0.004**	**1.560**	**0.004**	**1.573**	0.004	1.573
3DConv1	0.083	32.716	**0.006**	**2.378**	**0.006**	**2.416**	**0.001**	**0.566**	**0.003**	**1.265**	0.003	1.265	**0.003**	**1.302**	0.003	1.302
3DStack1_1	0.055	2.727	**0.003**	**0.153**	**0.008**	**1.183**	**0.001**	**0.117**	**0.005**	**0.642**	0.005	0.642	**0.005**	**0.645**	0.005	0.645
3DStack2_1	0.111	5.442	**0.006**	**0.299**	**0.020**	**1.019**	**0.001**	**0.060**	**0.003**	**0.156**	0.003	0.156	**0.003**	**0.159**	0.003	0.159
3DStack3_1	0.111	0.681	**0.006**	**0.037**	**0.020**	**0.496**	**0.002**	**0.026**	**0.008**	**0.143**	0.008	0.143	**0.008**	**0.143**	0.008	0.143
3DStack4_1	0.111	0.681	**0.006**	**0.037**	**0.020**	**0.127**	**0.001**	**0.007**	**0.003**	**0.019**	0.003	0.019	**0.003**	**0.020**	0.003	0.020
3DStack5_1	0.111	5.442	0.111	5.442	0.111	5.442	0.111	5.442	0.111	5.442	0.111	5.442	0.111	5.422	**0.003**	**0.159**
3DStack6_1	0.055	21.768	0.055	21.768	0.055	21.768	0.055	21.768	0.055	21.768	0.055	21.768	0.055	21.768	**0.002**	**0.755**
3DStack1_2	0.055	2.727	**0.003**	**0.153**	**0.008**	**1.183**	**0.001**	**0.117**	**0.005**	**0.642**	0.005	0.642	**0.005**	**0.645**	0.005	0.645
3DStack2_2	0.111	5.442	**0.006**	**0.299**	**0.020**	**1.019**	**0.001**	**0.060**	**0.003**	**0.156**	0.003	0.156	**0.003**	**0.159**	0.003	0.159
3DStack3_2	0.111	0.681	**0.006**	**0.037**	**0.020**	**0.496**	**0.002**	**0.026**	**0.008**	**0.143**	0.008	0.143	**0.008**	**0.143**	0.008	0.143
3DStack4_2	0.111	0.681	**0.006**	**0.037**	**0.020**	**0.127**	**0.001**	**0.007**	**0.003**	**0.019**	0.003	0.019	**0.003**	**0.020**	0.003	0.020
3DStack5_2	0.111	5.442	0.111	5.442	0.111	5.442	0.111	5.442	0.111	5.442	0.111	5.442	0.111	5.422	**0.003**	**0.159**
3DStack6_2	0.055	21.768	0.055	21.768	0.055	21.768	0.055	21.768	0.055	21.768	0.055	21.768	0.055	21.768	**0.002**	**0.755**
3DStack1_3	0.055	2.727	**0.003**	**0.153**	**0.008**	**1.183**	**0.001**	**0.117**	**0.005**	**0.642**	0.005	0.642	**0.005**	**0.645**	0.005	0.645
3DStack2_3	0.111	5.442	**0.006**	**0.299**	**0.020**	**1.019**	**0.001**	**0.060**	**0.003**	**0.156**	0.003	0.156	**0.003**	**0.159**	0.003	0.159
3DStack3_3	0.111	0.681	**0.006**	**0.037**	**0.020**	**0.496**	**0.002**	**0.026**	**0.008**	**0.143**	0.008	0.143	**0.008**	**0.143**	0.008	0.143
3DStack4_3	0.111	0.681	**0.006**	**0.037**	**0.020**	**0.127**	**0.001**	**0.007**	**0.003**	**0.019**	0.003	0.019	**0.003**	**0.020**	0.003	0.020
3DStack5_3	0.111	5.442	0.111	5.442	0.111	5.442	0.111	5.442	0.111	5.442	0.111	5.442	0.111	5.422	**0.003**	**0.159**
3DStack6_3	0.055	21.768	0.055	21.768	0.055	21.768	0.055	21.768	0.055	21.768	0.055	21.768	0.055	21.768	**0.002**	**0.755**
Out1_1	0.028	10.91	**0.002**	**0.793**	**0.002**	**0.805**	**0.0004**	**0.189**	**0.001**	**0.422**	0.001	0.422	**0.001**	**0.434**	0.001	0.434
Out2_1	0.001	0.340	0.001	0.340	0.001	0.340	0.001	0.340	0.001	0.340	0.001	0.340	0.001	0.340	0.001	0.340
Out1_2	0.028	10.91	**0.002**	**0.793**	**0.002**	**0.805**	**0.0004**	**0.189**	**0.001**	**0.422**	0.001	0.422	**0.001**	**0.434**	0.001	0.434
Out2_2	0.001	0.340	0.001	0.340	0.001	0.340	0.001	0.340	0.001	0.340	0.001	0.340	0.001	0.340	0.001	0.340
Out1_3	0.028	10.91	**0.002**	**0.793**	**0.002**	**0.805**	**0.0004**	**0.189**	**0.001**	**0.422**	0.001	0.422	**0.001**	**0.434**	0.001	0.434
Out2_3	0.001	0.340	0.001	0.340	0.001	0.340	0.001	0.340	0.001	0.340	0.001	0.340	0.001	0.340	0.001	0.340
3D CNNs	1.887	198.470	0.631	110.766	0.772	117.737	0.571	106.194	0.619	109.842	0.568	89.621	0.568	89.728	0.085	10.840
Full Model	5.227	256.661	3.971	168.957	4.112	175.928	3.912	164.385	3.959	168.033	3.908	147.812	3.908	147.919	3.425	69.031
